# Global Trends of Artificial Intelligence Applications in Family Medicine: A Comprehensive Scopus-Based Bibliometric Analysis (2016–2026)

**DOI:** 10.3390/healthcare14213611

**Published:** 2026-10-26

**Authors:** Muhammed Fatih Baran, Ömer Adil İlhan

**Affiliations:** 1 Department of Family Medicine, Konya City Hospital, 42020 Konya, Turkey; 2 Family Medicine, Konya Meram State Hospital, 42090 Konya, Turkey; omeradil.ilhan@saglik.gov.tr

**Keywords:** artificial intelligence, family medicine, primary healthcare, bibliometric analysis, machine learning, Scopus

## Abstract

**Aim:** To map recent artificial intelligence research in family medicine and primary care against an earlier 2015–2024 bibliometric baseline. **Methods:** Scopus was searched on 9 July 2026 for 2016–2026 publications. One researcher screened 3452 articles and reviews; 132 topical and 13 language exclusions left 3307 records. Manual title, DOI, and Scopus-identifier checks identified no duplicates. VOSviewer 1.6.21 and Biblioshiny (Bibliometrix 5.4.1; R 4.6.1) supported descriptive analysis and mapping. **Results:** Output increased from 531 in 2024 to 792 in 2025 (49.2%). The 2025 and partial 2026 component contained 1407 records (42.5%); 615 were dated 2026. The USA led publication and raw citation rankings (711 publications; 18,671 citations), while China preceded the United Kingdom in publication count. The *Journal of Medical Internet Research* (78 publications) and *Scientific Reports* (69) led the venues; seven top ten venues were shared with the earlier study. Language model terminology coexisted with later-positioned explainability, risk stratification, and diagnostic accuracy terms. **Conclusions:** This updated map quantifies post-2024 activity and identifies continuity and corpus-dependent differences in geography, dissemination, and evaluation-related vocabulary. Single-database and English language coverage, single-researcher screening, unadjusted citations, and incomplete 2026 coverage limit inference. The findings describe research activity, not clinical adoption, safety, effectiveness, or causality.

## 1. Introduction

Artificial intelligence (AI) has become an active area of medical research, including image interpretation, prediction, and decision support [1,2]. Large language models (LLMs) have also been evaluated on clinical knowledge and medical examination tasks [3,4]. Performance on selected tasks should be distinguished from effectiveness in routine clinical care. For a rapidly expanding literature, an informative map needs to describe both the scale of recent activity and its organization: who produces the research, where it is disseminated, and which terms frame questions about model use and evaluation.

Primary care provides first-contact, comprehensive, continuous, and coordinated clinical care [5]. Family medicine is the medical discipline that commonly delivers these services. Primary healthcare is a broader concept that also encompasses community-oriented prevention and population health activities. In this study, these terms were included together as related search concepts, rather than treated as identical disciplines or service models. Records were intended to be relevant to AI in a family medicine or primary care context. This broad operational scope improves retrieval across differences in terminology but may also introduce variation in how directly publications address clinical family medicine. Multimorbidity and the time required for clinical and administrative work provide relevant context for studying digital support in these settings [6,7].

Relevant applications include clinical decision support [8]. Established cardiovascular prediction tools provide additional clinical context for risk assessment in primary care [9], although QRISK3 uses conventional regression rather than machine learning. Other AI applications include image-based screening [10,11] and natural language processing of electronic health records [12]. Generative AI research includes comparisons of chatbot and physician responses to patient questions [13], while telehealth research describes changes in the delivery of primary care visits [14]. These examples motivate mapping the research literature but do not demonstrate that AI tools are routinely deployed or clinically beneficial across primary care.

Bibliometric analysis describes publication patterns, citation distributions, collaboration structures, and relationships between research terms [15]. VOSviewer and Bibliometrix provide complementary tools for these descriptive tasks [16,17]. Such analyses complement clinical evidence syntheses but do not assess the methodological quality, safety, or effectiveness of individual AI interventions. Their findings also depend on database coverage, search terms, record selection, and data preparation.

Wang et al. provided an important baseline by mapping 653 Web of Science Core Collection publications from 2015 to 2024, including countries, institutions, journals, authors, and themes [18]. Their analysis already included LLM-related material. The remaining question is how the research landscape is characterized when substantial later-indexed activity is considered through an independently retrieved corpus. This study addresses that question in three ways: it quantifies the post-2024 component while separating the complete year 2025 from incomplete 2026; it identifies points of agreement and differences in reported geographic and journal profiles relative to the published baseline; and it interprets language model terminology together with explainability and model-evaluation terms rather than only listing clinical application areas. The Scopus query explicitly names natural language processing, neural networks, LLMs, and ChatGPT as related retrieval concepts. Its contribution is an updated, comparative, evaluation-oriented account of research activity. Differences in source coverage [19] and non-identical queries preclude treating the two corpora as interchangeable samples or claiming that greater size alone establishes superior completeness or quality.

Four research questions guide this descriptive study. RQ1: How did output change during the complete years 2016–2025, and how much of the retrieved corpus is dated after the earlier study’s 2024 cutoff, with 2026 reported separately? RQ2: Which countries, institutional labels, and journals lead the reported publication and raw citation outputs, and what author production patterns are displayed? RQ3: Which geographic and publication-venue findings agree with or differ from the published 2015–2024 profile, and what international collaboration associations are visible? RQ4: How are LLM-related concepts and terms concerning explainability, risk stratification, and diagnostic evaluation represented in the keyword and temporal outputs? These questions concern research organization and vocabulary; they do not test clinical effectiveness or causal effects of generative AI.

## 2.  Materials and Methods

### 2.1. Study Design and Data Source

This was a descriptive bibliometric study using Scopus as the sole source for the final dataset. The search snapshot was obtained on 9 July 2026 and covered records assigned to publication years 2016–2026. A preliminary database exploration had reported 1230 Web of Science records and 5061 Scopus records after a priori preprint exclusion. These exploratory totals were used for database selection only: they were not pooled, and the Web of Science set did not contribute to the final analysis. Scopus was selected to provide a single, internally consistent source of bibliographic and citation metadata across the selected literature. Known differences in journal coverage between Scopus and Web of Science support treating their outputs as database-dependent perspectives [19], rather than as directly comparable measures of retrieval quality.

PubMed, Web of Science, and other sources were not combined with Scopus. This decision maintained a common metadata structure and citation source and avoided introducing cross-database record matching and citation harmonization problems. It should not be interpreted as a claim that PubMed cannot support bibliometric analyses. The resulting landscape is limited to records retrieved from Scopus, and relevant publications unique to other sources may have been missed. The full topical query entered in Scopus was: TITLE-ABS-KEY ((“family medicine” OR “family practice” OR “primary care” OR “primary healthcare” OR “primary health care” OR “general practice” OR “general practitioner*”) AND (“artificial intelligence” OR “machine learning” OR “deep learning” OR “neural network*” OR “natural language processing” OR “large language model*” OR “ChatGPT”)). The subsequent publication year, document type, relevance, and language restrictions are described below. The explicit method and language model terms allow records indexed with those labels to match even when a generic AI label is absent from the searched fields; this is a feature of the query design, not a measured advantage in comparative recall or precision.

Records were exported from Scopus in CSV format with bibliographic and citation information, including authors, titles, publication years, source titles, abstracts, DOIs, Scopus identifiers, affiliation addresses, citation totals, Author Keywords, Index Keywords, and cited references. The cited reference field supplied the input for the co-citation analyses.

All reported numerical results refer to the original 9 July 2026 analysis snapshot (*n* = 3307). A subsequently refreshed export was used during revision to clarify the export structure, not to replace or update the original numerical outputs. The revision checked agreement between the text, archived tables, figures, and selection arithmetic; it did not independently recompute the original rankings from a later metadata version. This distinction is relevant because indexing, reference parsing, citation totals, and record versions can change between exports.

### 2.2. Inclusion and Exclusion Criteria

**Figure 1:**
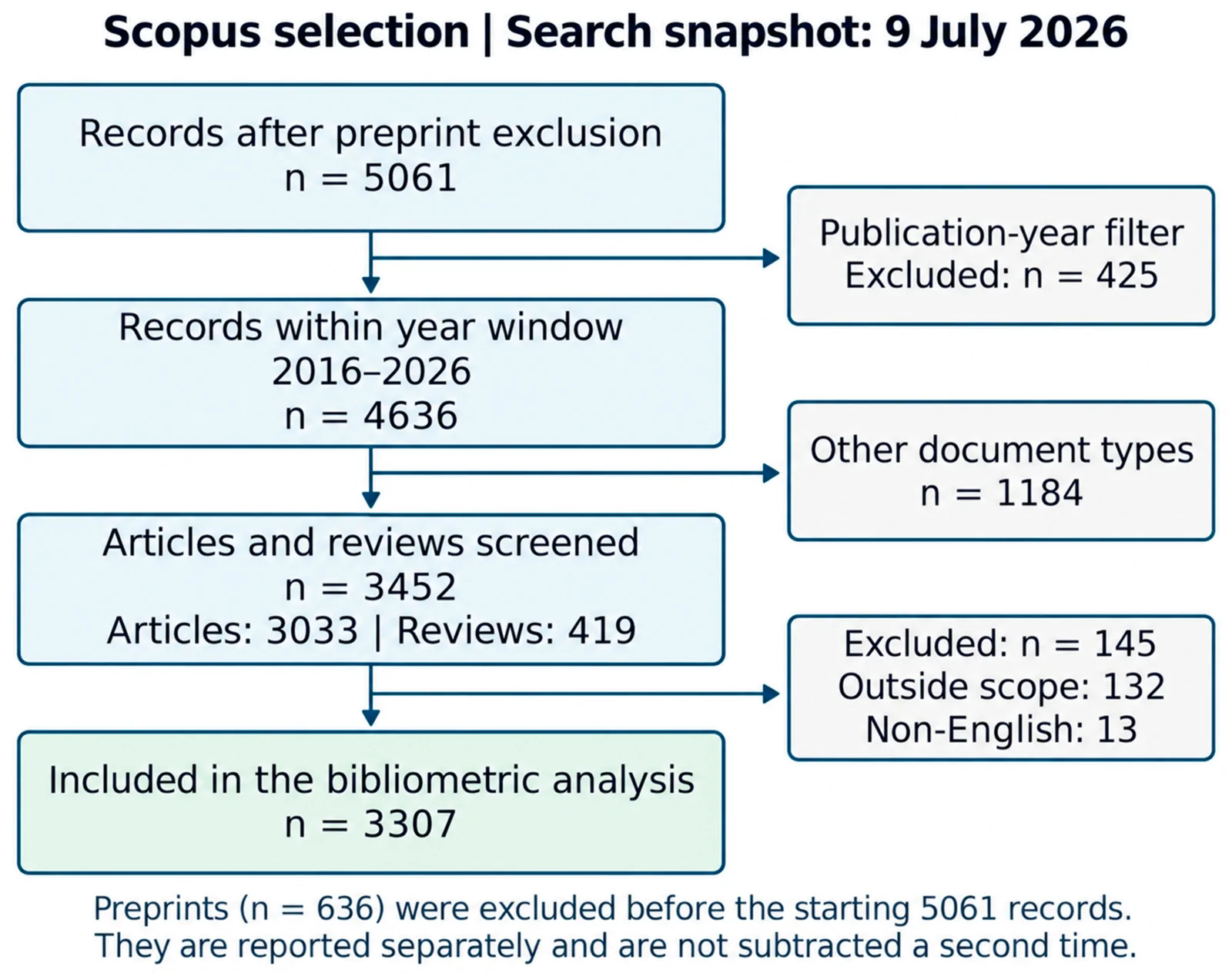
Scopus record selection flow for the 9 July 2026 snapshot. The starting 5061 records already exclude the separately reported 636 preprints. Exclusion branches reconcile retained totals; 425 and 1184 are differences between successive totals. Duplicate checking during dataset preparation identified no duplicates (0 removed). The exploratory Web of Science set is not included in this flow. Vertical arrows indicate the sequential record-selection process, while horizontal arrows indicate excluded records. Blue boxes represent successive selection stages, gray boxes indicate exclusions, and the green box represents the final dataset included in the analysis.

The study window comprised ten complete publication years (2016–2025) plus the incomplete 2026 retrieval snapshot. Figure 1 begins with the 5061 Scopus records already remaining after the reported a priori preprint exclusion (*n* = 636); those preprints are not subtracted again from 5061. Publication year filtering retained 4636 records, a reduction of 425. Restricting document types to Article (*n* = 3033) and Review (*n* = 419) retained 3452 records, a further reduction of 1184 for other document types. Article and Review are database document categories, not guarantees of methodological quality or a high level of clinical evidence.

Titles and abstracts were assessed by one researcher, Muhammed Fatih Baran, for relevance to both AI and the family medicine/primary care context. Screening excluded 132 records outside this combined topical scope and 13 non-English records, leaving 3307 publications (3452 − 132 − 13). During preparation of this original analysis dataset, duplication was checked manually by comparing titles, DOIs, and Scopus record identifiers (EIDs). No duplicate records were identified, and none were removed for duplication. This record-level check is distinct from the handling of author, affiliation, and keyword variants. The counts 425 and 1184 are arithmetic differences between successive retained totals. Figure 1 presents a bibliometric record selection diagram; it does not imply a clinical systematic review or full PRISMA compliance.

### 2.3. Data Analysis and Visualization

VOSviewer version 1.6.21 (Centre for Science and Technology Studies, Leiden University, Leiden, The Netherlands) [16] was used for network mapping. Biblioshiny, the web interface to the Bibliometrix package (version 5.4.1; developed by Massimo Aria and Corrado Cuccurullo, Italy) [17], was run in R version 4.6.1 (R Foundation for Statistical Computing, Vienna, Austria; 24 June 2026, ucrt) for descriptive summaries, author production over time, and trend topics. The study integrates descriptive performance analysis and exploratory science mapping [15]. The four network outputs represent different relationships: country co-authorship, cited-source/journal co-citation, cited-author co-citation, and keyword co-occurrence. Co-citation links describe joint citation in reference lists, not direct collaboration between cited researchers.

Full counting was used for the VOSviewer analyses. Minimum threshold entries were 5 publications per country for the co-authorship network, 100 citations per cited source for journal co-citation, 50 citations per cited author label for author co-citation, and 20 occurrences per keyword label for co-occurrence. These are inclusive minimum thresholds, not strict greater-than cutoffs. Different thresholds were selected to reduce visual crowding and focus interpretation on repeatedly represented entities; no claim of an empirically optimal threshold or sensitivity-tested cluster stability is made. Default normalization, layout, and clustering options were not changed: association-strength normalization and clustering resolution 1.00 were retained. Default attraction was 2; repulsion was 1 for the country and co-citation maps, and 0 for keyword co-occurrence. Other advanced layout and clustering options remained at their software defaults.

Country attribution used affiliation addresses of all authors rather than the corresponding author’s address alone. With full counting, a publication contributes to each represented country, so country totals need not sum to the corpus size. Institutional labels were taken from the original affiliation outputs without manual name merging or campus/system consolidation; the institutional analysis presents descriptive rankings rather than a network requiring a separate map threshold.

Biblioshiny used Author Keywords and Index Keywords together for the term analyses, with minimum frequency 20 for the trend topic output. The direct author-production output was generated for the ten displayed indexed author labels. No additional manual citation-rate calculation was applied to this output. Its TC per Year indicator sums the publications’ accumulated citation counts divided by (analysis year − publication year + 1) within each author/publication year group; the analysis year was 2026. Thus, it is an annualized accumulated citation indicator, and does not comprise citations received during the displayed calendar year and not a field-normalized impact score.

Publication counts refer to bibliographic record counts in the reported entity-level outputs; citation counts refer to accumulated database citation totals in the original source snapshot, not to patient outcomes or effect sizes. Publication and citation rankings are presented separately. Raw citation totals are not adjusted for publication age, field, self-citation, or the concentration of citations in individual papers, and are not direct measures of quality or clinical influence. Keyword frequencies describe individual displayed labels, not mutually exclusive categories or unique topic prevalence. Association-strength normalization concerns network link weights; it does not normalize the raw citation totals in the tables.

Annual comparisons focus on the complete years 2016–2025. Records dated 2026 are reported separately and are not annualized or used to infer a decline relative to 2025. The post-2024 component comprises 2025 plus the incomplete 2026 snapshot. Its share is calculated against the final corpus; the 2024–2025 percentage change uses the 2024 count as denominator. These are descriptive arithmetic summaries. No exponential model, causal analysis, or inferential test was used.

Manual record-level duplicate checking used titles, DOIs, and Scopus identifiers. Entity and term labels were interpreted separately from that check. The retained outputs contain initial-based author names, cited source abbreviations, and singular/plural keyword variants; institutional names were not manually merged. No retrospective thesaurus harmonization was applied to the retained maps during revision. Variant frequencies were not added as if they represented distinct publications, because a record can contain more than one variant. Network clusters and temporal summaries were treated as exploratory outputs that depend on data preparation, thresholds, and mapping settings.

For contextual interpretation, the reported geographic and publication-venue profiles were compared with the published aggregate results of Wang et al. [18]. Journal-list overlap is the number of shared titles in the two reported publication top tens, allowing for transparent name abbreviations. The comparison describes agreement and differences in list membership and ordering; citation totals were not harmonized across sources. No pooled corpus, record-level overlap estimate, matched database experiment, or causal attribution of rank differences was undertaken.

## 3. Results

### 3.1. Distribution of Publications by Year and Growth Trend

**Figure 2:**
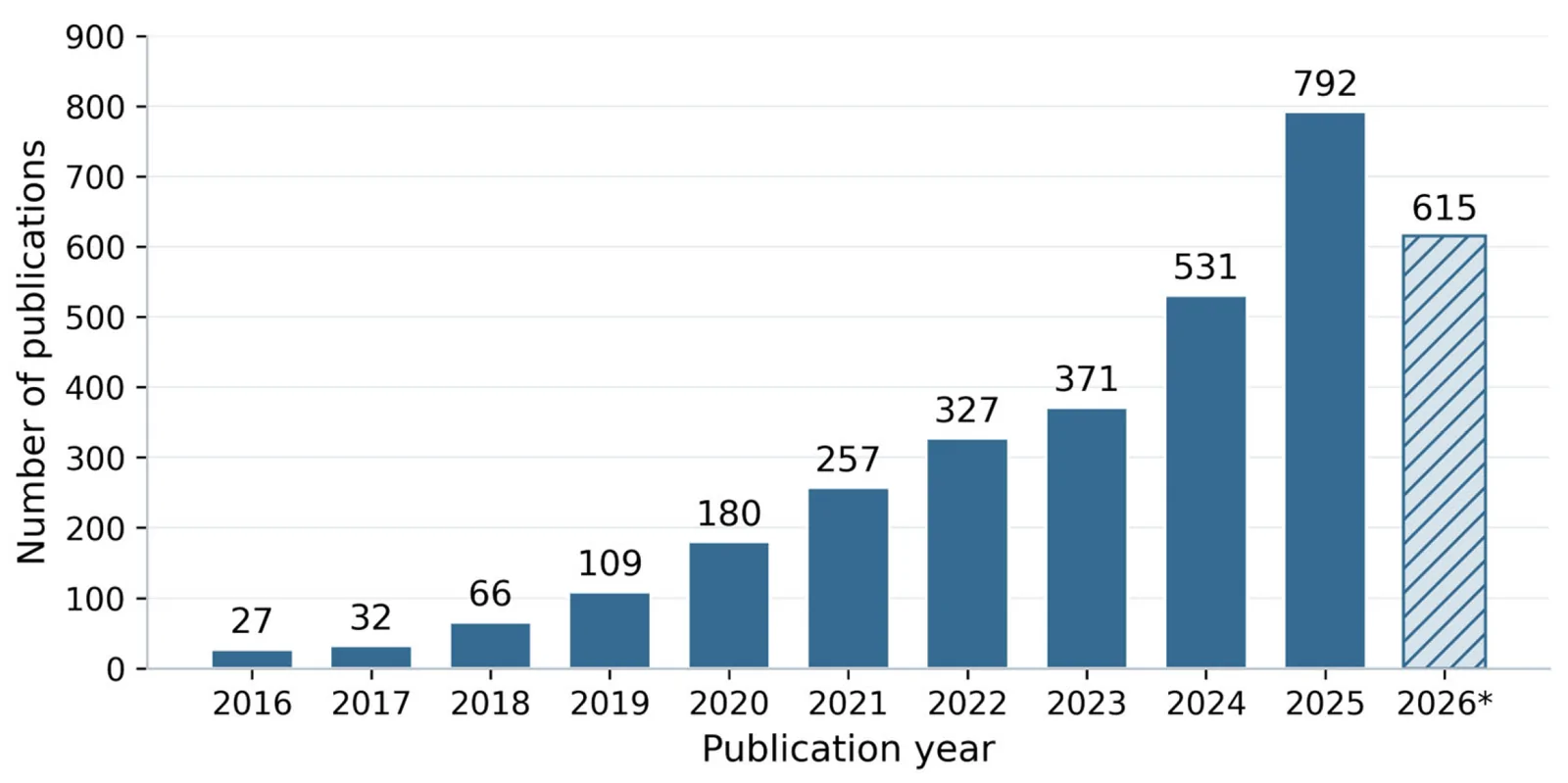
Observed annual publication counts. Years 2016–2025 are complete publication years; the hatched 2026 bar is the incomplete snapshot retrieved on 9 July 2026. No fitted growth curve or full-year extrapolation is shown.

The 3307 publications were distributed across 2016–2026 as shown in Figure 2. Output increased from 27 in 2016 to 792 in 2025, the last complete publication year. The increase from 531 in 2024 to 792 in 2025 was 261 publications (49.2%). The complete years 2016–2025 contributed 2692 records. The 615 records dated 2026 were available at retrieval on 9 July 2026 and represent incomplete year coverage. Together, 2025 and the partial 2026 period contributed 1407 records (42.5% of 3307), whereas 1900 were dated 2016–2024. These totals quantify the recent component of this corpus; they do not identify records uniquely absent from the earlier study. No annualized projection, fitted exponential model, or causal test was performed, and no fitted trendline is shown.

### 3.2. General Distribution of Publications and Country Analysis

**Figure 3:**
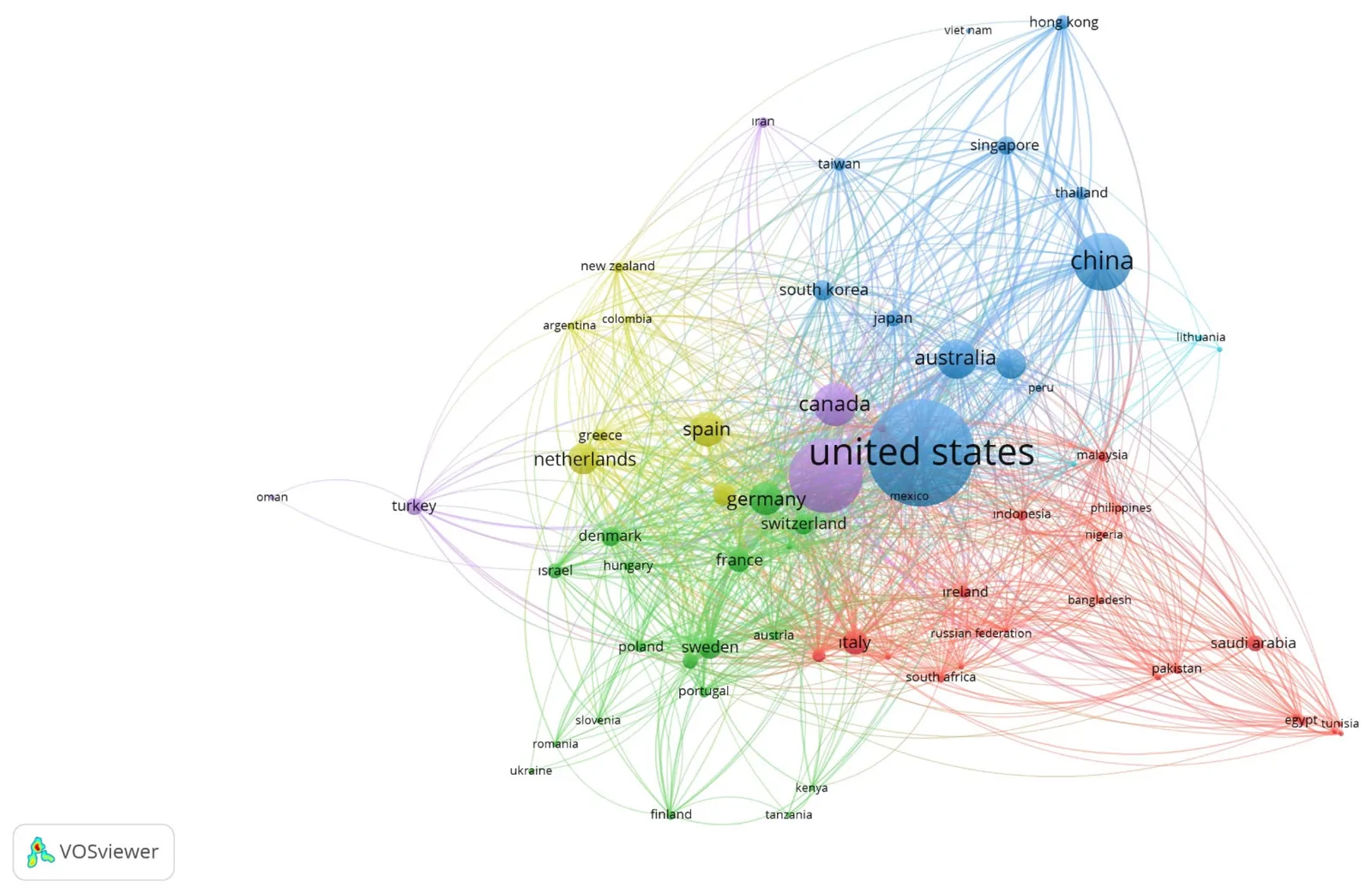
Country co-authorship network based on all authors’ affiliation addresses, using full counting and a minimum of 5 publications per country. Links indicate co-authorship across countries; colors identify clusters obtained with association-strength normalization and default clustering resolution 1.00. Visual prominence is not a formal centrality estimate. Node size reflects the relative publication output of each country, links indicate co-authorship relationships, and colors denote network clusters identified by VOSviewer.

Table 1 separates publication and raw citation rankings. The USA led both (711 publications; 18,671 citations). China (373) preceded the United Kingdom (368) in publication count, while the United Kingdom was second in raw citations (8017). Figure 3 shows a prominent USA node with international links. These indicators do not measure clinical influence, research quality, or formal network centrality.

### 3.3. Institutional Productivity and Citation Counts

The University of Toronto (146 publications) and the University of California (117) led the reported institutional publication ranking (Table 2). Mayo Clinic (1436 citations) and Harvard Medical School (1379) led the separate raw citation ranking. These lists are independently ordered. Affiliation labels were retained as represented in the original outputs, without manual institutional name consolidation. Labels may represent different organizational levels, such as a university system, campus, or hospital. The University of California label does not identify a single campus, and the abbreviated Norwegian University label is not assigned a more specific identity. These limitations prevent treating the list as a fully standardized comparison of institutions. Raw citation totals are not measures of institutional clinical performance.

### 3.4. Journal (Source) Analysis

**Figure 4:**
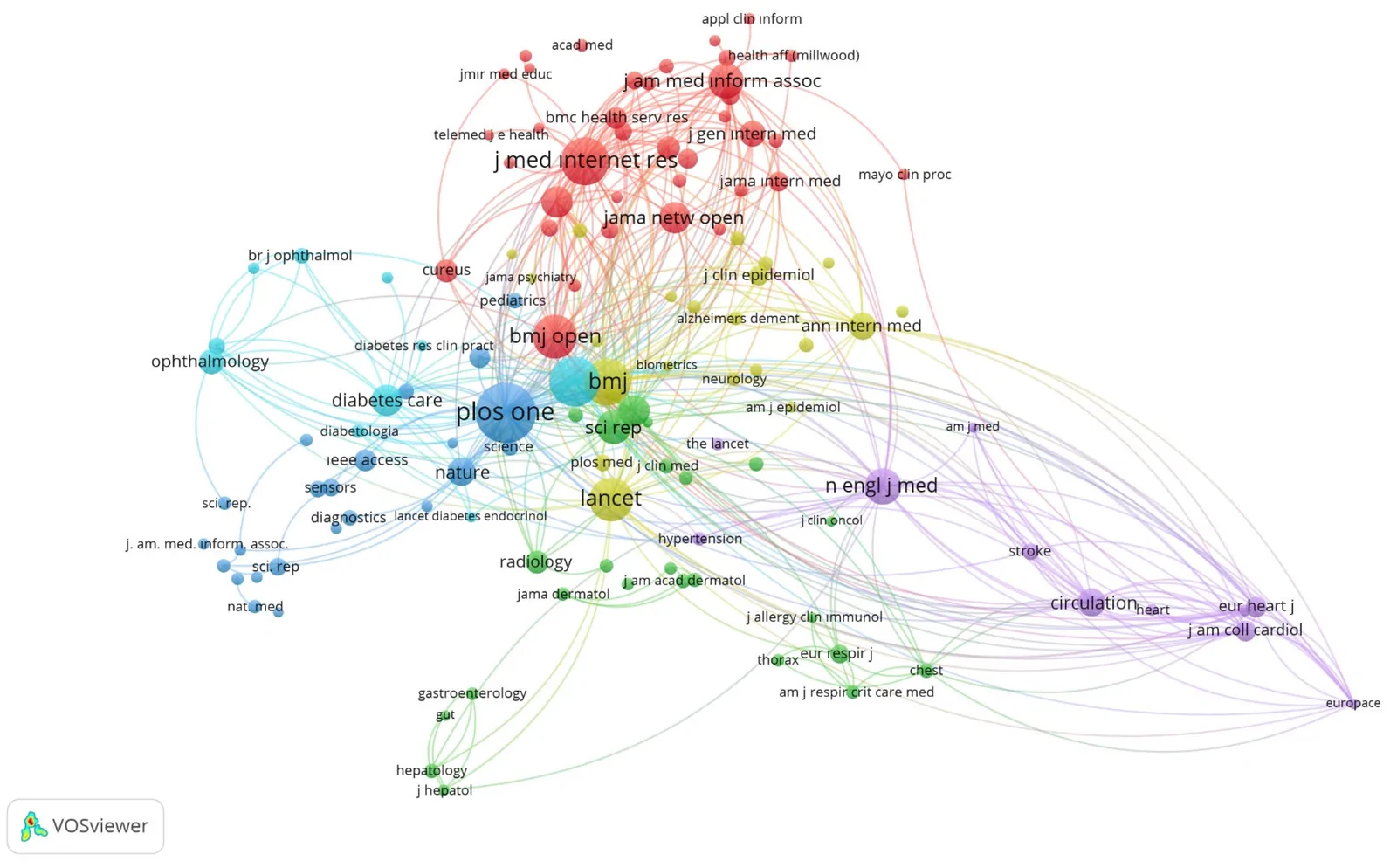
Cited-source (journal) co-citation network, using full counting, a minimum of 100 citations per source, association-strength normalization, and default clustering resolution 1.00. Nodes represent cited journals, with node sizes reflecting their relative citation weights. Links indicate co-citation relationships, while colors distinguish clusters of closely related journals. Abbreviation variants remain separate in the retained output, which is distinct from the publication-venue ranking in Table 3.

The *Journal of Medical Internet Research* (78 publications), *Scientific Reports* (69), *PLOS ONE* (63), and *BMJ Open* (56) led the publication ranking (Table 3). Seven journal titles were shared with the published top ten list of Wang et al. [18]. The *Journal of Medical Internet Research* ranked first here and fourth in that list, whereas *Scientific Reports* ranked second here and first there. *International Journal of Medical Informatics*, *Frontiers in Medicine*, and *JAMA Network Open* appeared in the current top ten but not in the comparator’s publication top ten list. These are comparisons of reported lists from different corpora, not evidence of a temporal rank change under matched retrieval conditions. Figure 4 instead maps journal co-citation, a cited source relationship distinct from publication-venue frequency.

### 3.5. Author Productivity and Co-Citation Analysis

**Figure 5:**
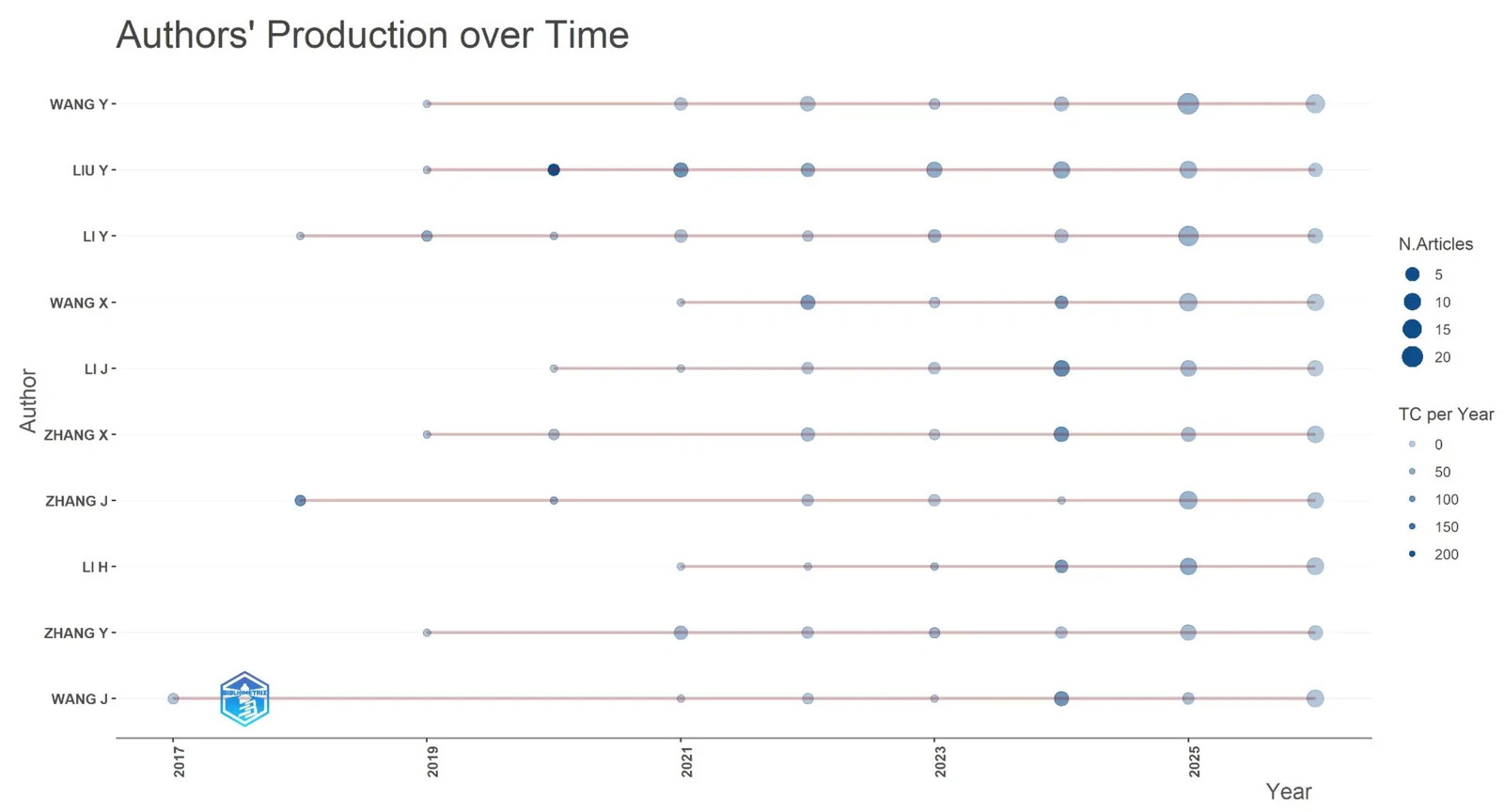
Direct Biblioshiny output of annual production for the ten displayed indexed author labels (Bibliometrix [17]). Red horizontal lines indicate the time span of publication activity for each indexed author label, connecting the years in which publications were recorded. Bubble size denotes publication count. Shading denotes TC per Year, the sum of annualized accumulated citation counts for each author’s publications from the displayed year, calculated as TC/(2026 − publication year + 1) for each publication. This is not citations received during that calendar year or a field-normalized impact measure. The 2026 output is incomplete; author initials may be affected by homonymy.

**Figure 6:**
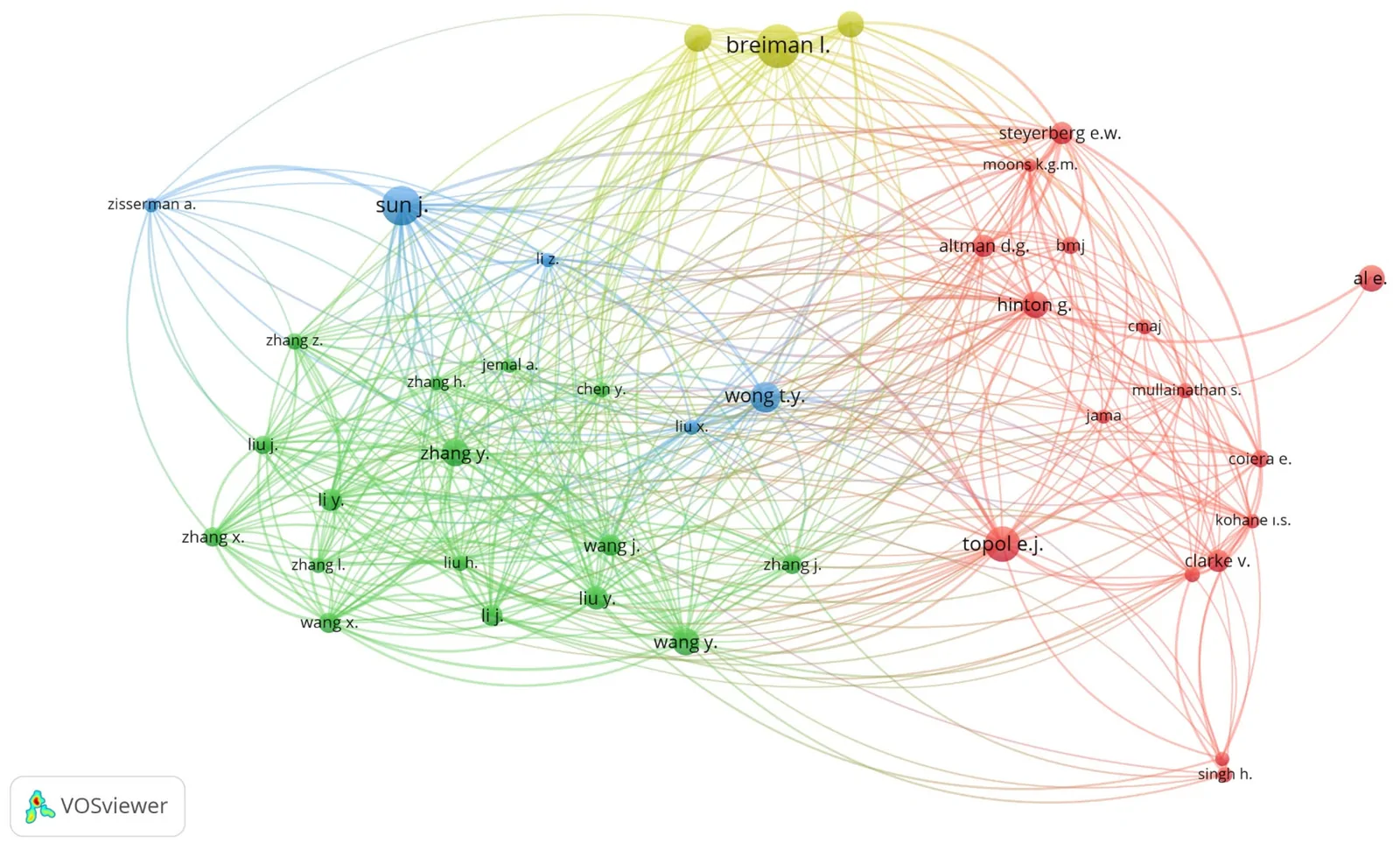
Cited-author co-citation network generated in VOSviewer using full counting and a minimum of 50 citations per cited-author label. Association-strength normalization and default clustering resolution 1.00 were retained. Nodes represent cited-author labels, with node sizes reflecting their relative citation weights. Colors indicate distinct clusters identified by VOSviewer. Links represent co-citation relationships between cited-author labels, with thicker links indicating stronger co-citation connections. These relationships reflect joint citation in reference lists rather than direct co-authorship.

Figure 5 is the direct Biblioshiny author production output for ten indexed labels: Wang Y., Liu Y., Li Y., Wang X., Li J., Zhang X., Zhang J., Li H., Zhang Y., and Wang J. Bubble size represents the number of publications attributed to a label in each publication year; shading represents the software-calculated TC per Year indicator, as defined in the Methods. Abbreviated labels may combine homonymous researchers. Figure 6 is a cited author co-citation map: the VOSviewer analysis type was co-citation and the unit was cited authors. Links indicate that cited author labels occur together in reference lists. Figure 6 describes co-citation relationships among cited author labels and does not represent direct co-authorship.

### 3.6. Keyword and Trending Topic Analysis

Table 4 reports frequencies for the term labels as displayed in the original keyword output. Artificial intelligence (826) and machine learning (609) were the most frequent labels. Other labels included telemedicine (72), digital health (71), large language models (64), clinical decision support (48), ChatGPT (46), and risk prediction (38). Lexical variants such as large language model/large language models and electronic health record/electronic health records remain separate. Their frequencies must not be summed to estimate numbers of unique topic-specific publications, because one record may contain more than one variant.

**Figure 7:**
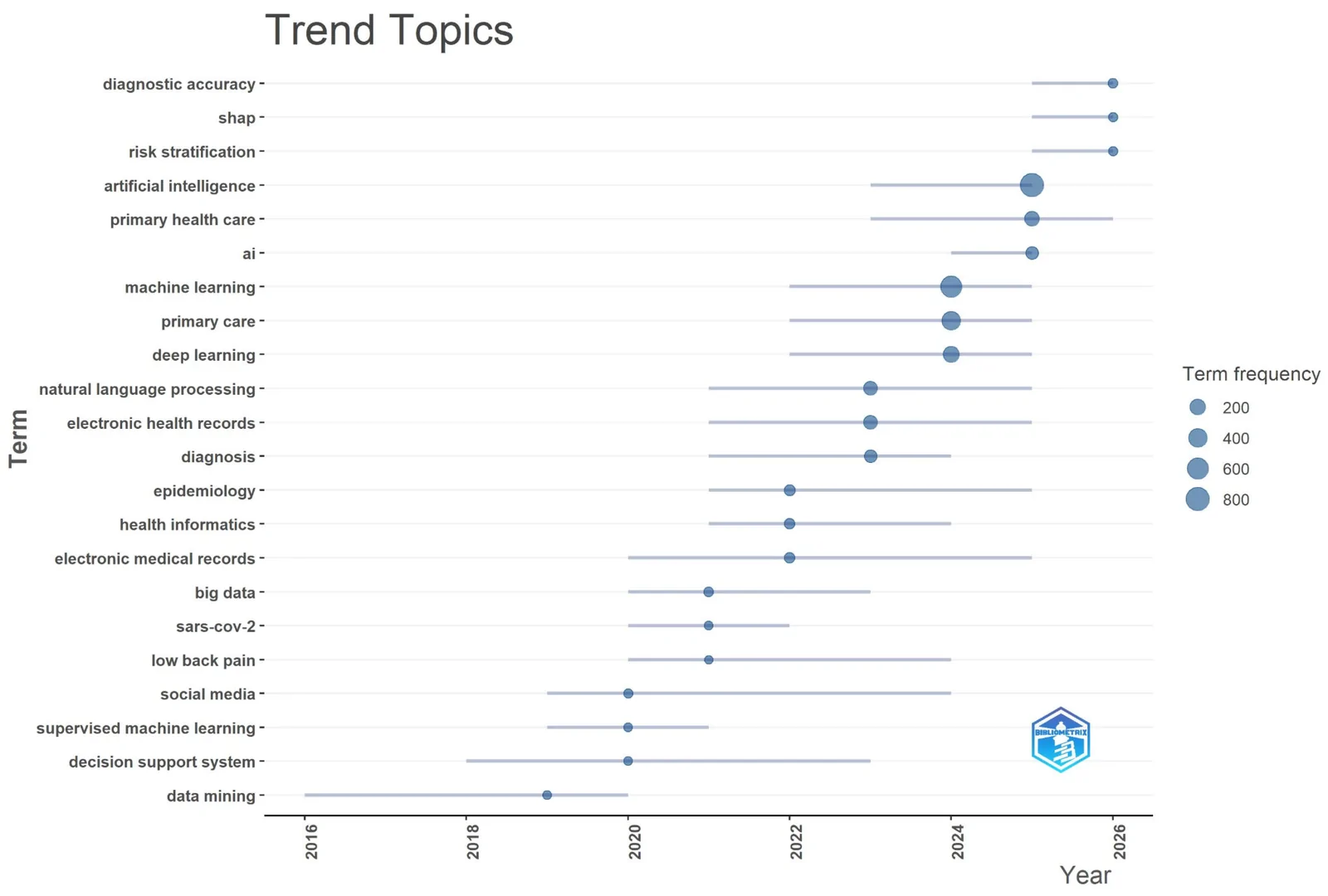
Biblioshiny trend-topic output using Author Keywords and Index Keywords together, with a minimum term frequency of 20. The horizontal axis represents publication year. Horizontal line segments indicate the interquartile range (Q1–Q3) of the temporal distribution of each term, while bubble positions represent the median publication year. Bubble size reflects the overall frequency of each term. Temporal summaries concern term use in the retrieved records; positions in the incomplete 2026 snapshot do not indicate dates of clinical adoption.

**Figure 8:**
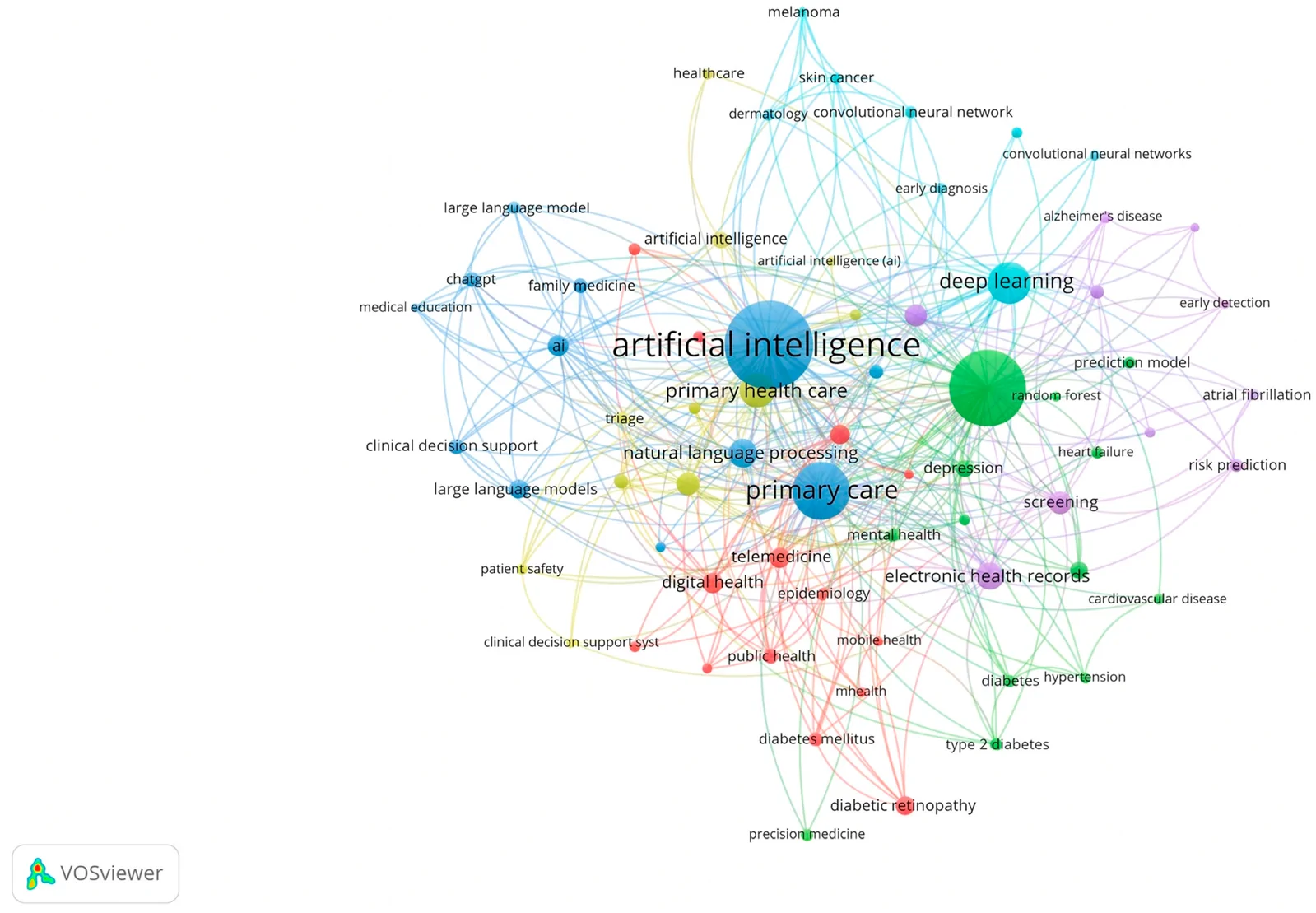
Keyword co-occurrence network generated using VOSviewer with full counting, a minimum of 20 occurrences per keyword label, association-strength normalization, and default clustering resolution 1.00. Nodes represent individual keyword labels, with node sizes reflecting their relative occurrence frequencies. Colors indicate clusters of related keywords identified by VOSviewer, while links represent keyword co-occurrence relationships within bibliographic records. Singular/plural and abbreviation variants remain separate; frequencies are not mutually exclusive clinical-topic counts.

Figure 7 displays the temporal positions of selected trend terms. Data mining is positioned earlier, while SHAP, risk stratification, and diagnostic accuracy occur toward the later end. SHAP denotes SHapley Additive exPlanations, an approach to explaining model predictions. These labels add an evaluation-related perspective to the general technology and application vocabulary. Temporal positions do not establish first appearance, implementation, or clinical effectiveness. ChatGPT and LLM-related labels are present in Table 4 and Figure 8, rather than in Figure 7. The co-occurrence map connects general AI and primary care terms with language models, image analysis, screening, prediction, and digital health terms; the visual groupings are exploratory bibliographic associations, not validated clinical categories.

## 4.  Discussion

The findings answer a more specific question than whether primary care AI research is growing. They describe a landscape with a substantial post-2024 component, points of agreement and differences in geographic and journal profiles relative to the earlier literature map, and a vocabulary that includes both language model technologies and questions of explanation and diagnostic evaluation. The 1407 records dated 2025 or from the partial 2026 period constitute 42.5% of the retrieved corpus. By relating this temporal component to where research is produced, disseminated, and discussed, the study provides an updated reference for planning evidence searches and research collaborations. Its observations concern research activity, not the clinical performance of AI systems.

The comparison with Wang et al. [18] makes the scope of the contribution explicit. That study mapped 2015–2024, while the present corpus covers 2016 through the 9 July 2026 snapshot. Our 1407 post-2024 records quantify material beyond its publication year window; they should not be called 1407 unique new studies without record matching. Even after those years are set aside, 1900 records remain in 2016–2024, compared with their 653 in 2015–2024. Calendar extension therefore cannot explain the entire size difference. The AI query blocks are non-identical: our query explicitly names neural networks, natural language processing, and LLM/ChatGPT terms, while the earlier query also includes named learning paradigms. Web of Science topic fields and Scopus TITLE-ABS-KEY are not identical. Moreover, Wang et al. applied full text screening after title/abstract screening; our reported eligibility assessment is at title/abstract and language level. These differences jointly define distinct retrieved corpora. Their individual contributions to the total cannot be separated without matched searches and record-level comparison. Broader medical AI and ChatGPT bibliometric studies provide context rather than interchangeable comparator datasets [20,21,22,23].

The geographic comparison distinguishes a finding shared by the two corpora from a detail that differs. The USA leads publication output in both studies, and the United Kingdom is second in raw citations in both. However, China precedes the United Kingdom in the current publication list, whereas their order is reversed in Wang et al. [18]. This is a corpus-dependent contrast, not proof that national activity changed because of the additional years alone. The prominent countries are consistent with the concentration of research infrastructure and digital health capacity discussed elsewhere [24,25]. Yet publication addresses do not identify model-training populations, quantify funding, or measure algorithmic bias. The findings can inform questions about international participation and transferability without establishing performance in any country or population.

The journal comparison similarly shows continuity alongside a different ordering: seven of the ten leading venues are shared, but the *Journal of Medical Internet Research* heads the present list, compared with *Scientific Reports* in the earlier study [18]. The three non-shared current venues broaden the reported dissemination profile across medical informatics, general medicine, and open clinical research. This offers a concrete reference for locating relevant literature; it does not establish a longitudinal migration of the field because database, period, and query all differ. The University of Toronto also leads publication volume in both studies, while the current institutional citation list is led by Mayo Clinic and Harvard Medical School. Publication volume, accumulated citations, and organizational labels therefore need separate interpretation. Citation totals and journal identity do not establish methodological quality. Author productivity and cited author co-citation answer different questions: the former describes output attributed to indexed labels, whereas the latter describes shared reference use. Initial-based names limit individual attribution; co-citation does not establish direct collaboration or scientific influence.

The thematic contribution is an evaluation-oriented reading of the later research vocabulary. Wang et al. [18] already discussed LLMs and mapped clinical application contexts; merely identifying ChatGPT is therefore not the novelty. In the current outputs, LLM/ChatGPT labels coexist with later-positioned terms concerning SHAP, risk stratification, and diagnostic accuracy. Reading these terms together directs attention to how predictions are explained, how risk groups are defined, and how diagnostic performance is evaluated, alongside what a model can generate. These are research priorities suggested by the vocabulary, not outcomes assessed in the included papers. Reliability and hallucination remain relevant to LLM evaluation [3,4,13,26,27]. SHAP explains model predictions [28], but explanation alone does not establish safe clinical decisions [29]. Apparent thematic differences may also reflect query wording, retained lexical variants, and incomplete coverage in 2026.

For family medicine researchers, the practical value of this map is to connect a current set of publication venues and potential collaboration settings with questions requiring evidence synthesis and clinical evaluation. Primary care and health system reviews describe development, workflow, and implementation challenges [30,31], while empirical studies illustrate risks of algorithmic bias [32,33]. Ethical governance and appropriate trial reporting are relevant when AI interventions are evaluated [34,35]. Problems identified in COVID-19 prediction model research illustrate that specific literature rather than the risk of bias of our corpus is needed [36]. TRIPOD + AI provides reporting guidance for prediction model studies [37]. These sources support priorities for subsequent research; the present metadata analysis did not appraise validation quality, safety, adoption, or patient outcomes.

Strengths include a clearly dated single-source snapshot, an explicit topical query, a manual duplicate check with no duplicates identified, reconciled selection totals, separation of complete and incomplete years, and complementary publication, citation, and term outputs. The contextual comparison identifies shared findings and differences rather than equating larger retrieval with greater quality. Several limitations remain. First, relevant publications unique to PubMed, Web of Science, or other sources may be absent, and source coverage can alter the observed profiles [19]. Second, the broad family medicine/primary care/primary healthcare scope includes records with varying direct relevance to clinical family medicine. Title/abstract screening by one researcher was not independently duplicated and may have missed eligible records or retained borderline topics. Third, English-only and Article/Review restrictions exclude other languages, conference outputs, and gray literature.

Fourth, 2026 coverage ends at the retrieval snapshot. Recent publications, especially those from 2025 to 2026, have shorter citation windows, whereas one or a few highly cited papers can dominate an entity’s citation total. Fifth, retaining affiliation labels without manual consolidation can fragment institutions or mix organizational levels. Author homonymy, reference parsing limitations, source abbreviations, and unmerged keyword variants can also affect the maps. Display thresholds reduce visual density but may exclude emerging low-frequency topics; default clustering settings do not establish cluster robustness. Sixth, the original numerical outputs were retained during revision rather than independently recomputed from a later refreshed export. Exact record-level reproduction depends on the original frozen export; later metadata versions can differ even when the total record count is unchanged. Finally, bibliometric metadata cannot establish methodological quality, clinical validity, adoption, effectiveness, safety, or causality.

## 5. Conclusions

This study adds a current Scopus-based perspective to the earlier primary care AI literature map. Among 3307 publications retrieved on 9 July 2026, 1407 (42.5%) were dated 2025 or to the incomplete 2026 period. Complete year output rose from 531 in 2024 to 792 in 2025; the 615 records dated 2026 were reported separately. The USA led publication and raw citation rankings, while China preceded the United Kingdom in publication count. The *Journal of Medical Internet Research* led the journal list; seven of the top ten venues were shared with the earlier map, showing continuity alongside corpus-dependent differences. LLM-related labels and later-positioned SHAP, risk stratification, and diagnostic accuracy terms provide an evaluation-oriented perspective for subsequent research. The contribution is a contextualized account of recent activity, dissemination, and vocabulary, subject to retrieval and citation limitations. It does not establish routine adoption, clinical validity, safety, or a causal effect of generative AI.

## Figures and Tables

**Table 1:** Separate country rankings by reported publication counts and raw citation totals.

Rank	Country	Publications	Country	Citations
1	USA	711	USA	18,671
2	CHINA	373	UNITED KINGDOM	8017
3	UNITED KINGDOM	368	CHINA	4903
4	CANADA	164	CANADA	3765
5	AUSTRALIA	139	AUSTRALIA	2546
6	SPAIN	118	KOREA	2195
7	INDIA	100	SINGAPORE	2169
8	GERMANY	94	NETHERLANDS	1855
9	NETHERLANDS	84	SPAIN	1419
10	ITALY	67	GERMANY	1371

**Table 2:** Separate institutional rankings by reported publication counts and raw citation totals.

Rank	Institution	Publications	Institution	Citations
1	UNIVERSITY OF TORONTO	146	MAYO CLINIC	1436
2	UNIVERSITY OF CALIFORNIA	117	HARVARD MEDICAL SCHOOL	1379
3	HARVARD MEDICAL SCHOOL	114	BRIGHAM AND WOMEN’S HOSPITAL	313
4	IMPERIAL COLLEGE LONDON	107	MCGILL UNIVERSITY	268
5	MAYO CLINIC	94	VANDERBILT UNIVERSITY	194
6	UNIVERSITY OF OXFORD	93	Massachusetts General Hospital	152
7	UNIVERSITY COLLEGE LONDON	70	UNIVERSITY OF TORONTO	146
8	STANFORD UNIVERSITY	66	NORWEGIAN UNIVERSITY	143
9	UNIVERSITY OF CALGARY	56	DUKE UNIVERSITY	131
10	UNIVERSITY OF MANCHESTER	55	UNIVERSITY COLLEGE LONDON	123

**Table 3:** Journals ranked by reported publication count, with raw citation totals for the study output.

Rank	Journal	Publications	Citations
1	*JOURNAL OF MEDICAL INTERNET RESEARCH*	78	1163
2	*SCIENTIFIC REPORTS*	69	694
3	*PLOS ONE*	63	1714
4	*BMJ OPEN*	56	1008
5	*INT. JOURNAL OF MEDICAL INFORMATICS*	48	248
6	*BMC MED. INFORMATICS AND DECISION MAK.*	38	353
7	*JMIR MEDICAL INFORMATICS*	38	245
8	*FRONTIERS IN MEDICINE*	36	209
9	*NPJ DIGITAL MEDICINE*	35	603
10	*JAMA NETWORK OPEN*	34	603

**Table 4:** Frequencies of individual keyword labels in the original study output; lexical variants are shown separately.

Term	Frequency	Term	Frequency
Artificial intelligence	826	Clinical decision support	48
Machine learning	609	ChatGPT	46
Primary care	392	Public health	45
Deep learning	226	Electronic health record	43
Primary healthcare	164	Family medicine	43
Natural language processing	121	Qualitative research	43
Electronic health records	114	Dementia	38
Screening	91	Risk prediction	38
General practice	84	Diabetes mellitus	35
Diagnosis	79	Convolutional neural network	33
AI	78	Type 2 diabetes	33
Telemedicine	72	Primary healthcare	32
COVID-19	71	General practitioners	31
Digital health	71	Large language model	31
Large language models	64	Diabetes	30
Diabetic retinopathy	62	Epidemiology	30
Prediction	56	Healthcare	30
Depression	51	Mental health	30

## Data Availability

Bibliographic metadata were retrieved from Scopus (Elsevier; https://www.scopus.com) on 9 July 2026. The query, filters, software settings, and selection totals are reported in the Methods and figure captions. Tables and figures retain the original analysis outputs (*n* = 3307); the refreshed export was not substituted for that snapshot. Exact record-level reproduction requires the original frozen export. Data access and redistribution are subject to Scopus licensing conditions. No individual patient data were used.
